# Pyomyositis associated with abscess formation caused by streptococcus pneumoniae in children: a case report and review of literature

**DOI:** 10.1186/s13052-023-01472-1

**Published:** 2023-06-14

**Authors:** Luca Barchi, Michele Fastiggi, Ilaria Bassoli, Federico Bonvicini, Monica Silvotti, Lorenzo Iughetti, Alessandro De Fanti

**Affiliations:** 1 School of Pediatric, University of Modena and Reggio Emilia, Modena, 41224 Italy; 2 Paediatrics Unit, Santa Maria Nuova Hospital, AUSL-IRCCS of Reggio Emilia, Reggio Emilia, 42123 Italy; 3 Unit of Radiology, IRCCS-Arcispedale Santa Maria Nuova, Reggio Emilia, 42123 Italy

**Keywords:** Children, Pyomyositis, Psoas abscess, Myositis, Streptococcus Pneumoniae

## Abstract

**Background:**

Pyomyositis is an unusual bacterial infection but potential severe in children. Staphylococcus Aureus is the main caused of this disease (70–90%), following by Streptococcus Pyogenes (4–16%). Streptococcus Pneumoniae rarely caused invasive muscular infections. We describe a case of pyomyositis caused by Streptococcus Pneumonia in an adolescent 12-year-old female.

**Case presentation:**

I.L. referred to our hospital for high fever associated with right hip and abdominal pain. The blood exams showed increase of leukocytes with prevalence of neutrophils with high level of inflammatory markers (CRP 46,17 mg/dl; Procalcitonin 25,8 ng/ml). The abdomen ultrasonography was unremarkable. The CT and MRI of the abdomen and right hip revealed pyomyositis of the iliopsoas, piriformis and internal shutter associated with collection of pus between the muscular planes (Fig. 1). The patient was admitted to our paediatric care unit, and she was initially treatment with intravenous Ceftriaxone (100 mg/kg/day) and Vancomycin (60 mg/kg/day). On day 2, a pansensitive Streptococcus Pneumoniae was isolated from the blood culture, and the antibiotic treatment was changed to only IV Ceftriaxone. She was successively treated with IV Ceftriaxone for 3 weeks, then continued with oral Amoxicillin for a total of 6 weeks of therapy. The follow up showed a complete resolution of the pyomyositis and psoas abscess after 2 months.

**Conclusion:**

Pyomyositis associate with abscess is a rare and very dangerous disease in children. The clinical presentation can mimic symptoms of other pathologies like osteomyelitis or septic arthritis, so many times is hard to identify. The main risk factors include story of recent trauma and immunodeficiency, not present in our case report. The therapy involves the antibiotics and, if possible, abscess drainage. In literature there is much discussion about duration of antibiotic therapy.

**Supplementary Information:**

The online version contains supplementary material available at 10.1186/s13052-023-01472-1.

## Background

Pyomyositis associated with a primary abscess is a rare disease. This disease is more frequent in tropical countries [1]; males are more commonly affected than female. Primary abscesses usually develop from hematogenous, percutaneous injury lesions or lymphatic sites. The main risk factors include state of immunosuppression, renal failure, malnutrition, diabetes and trauma [2, 3]. The most common pathogen is Staphylococcus aureus that caused about 70–90% of cases in the worldwide [1–4]. Less common pathogens include beta-haemolytic streptococci, gram-negative enteric bacilli and Streptococcus Pneumonia. The purpose of this review was to provide information on pyomyositis associated with abscesses caused by Streptococcus Pneumoniae in pediatric patients by examining the literature in this field. These data could help physicians to recognize the characteristics features, to determine the correct diagnostic approach and in deciding the appropriate therapy to decrease morbidity and mortality associated with this disease.

## Case presentation

**Fig. 1:**
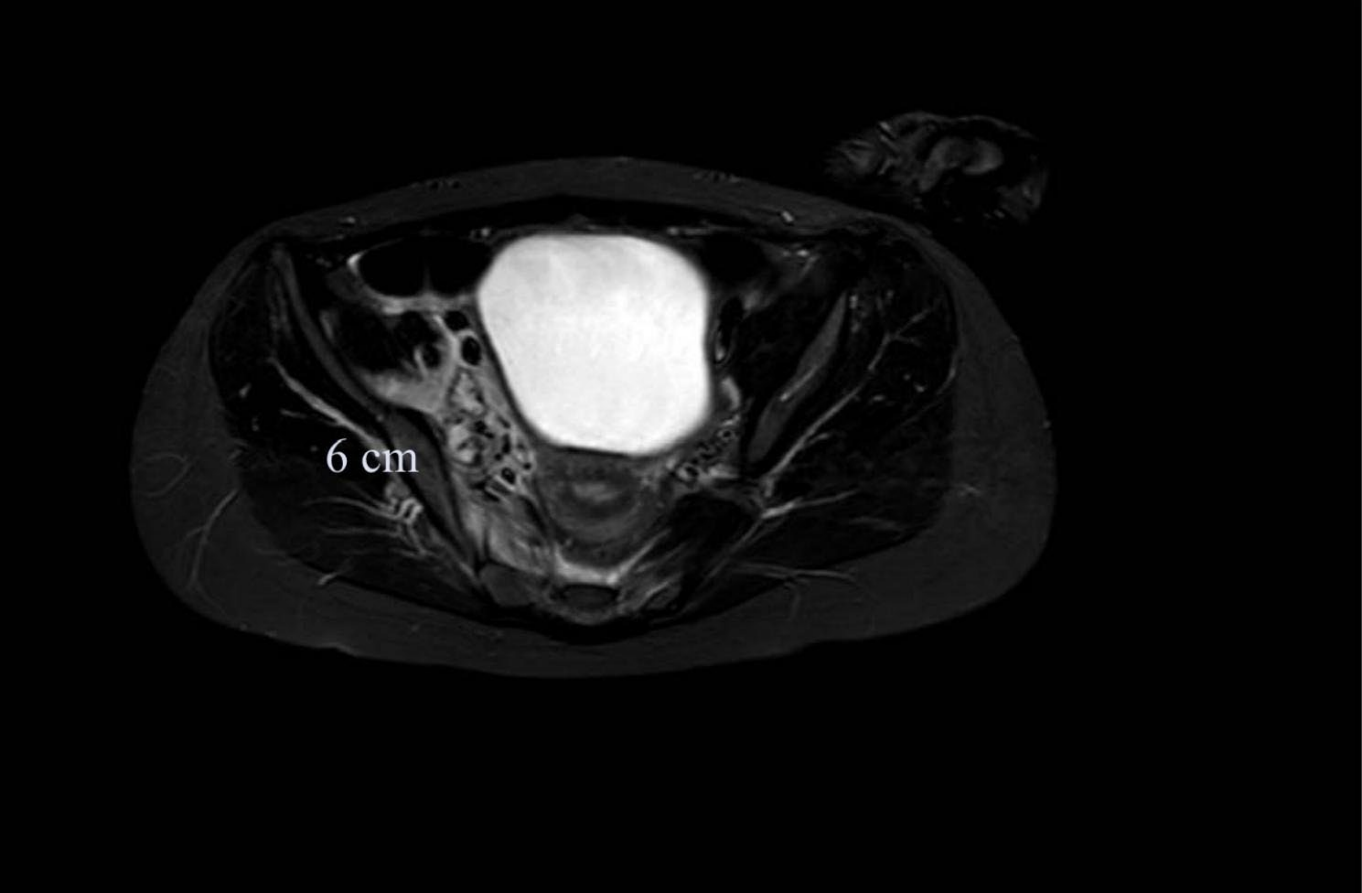
Short Time Inversion Recovery (STIR) MRI. The imagine shows edema of iliopsoas, piriformis and internal shutter. Between muscles there are multiple bunches (6 × 3,5 cm)

A previously healthy 12 year old female was hospitalized to community hospital with a history of 5 days of fever, cough and right hip and abdominal pain. Medical history was unremarkable and there was not history of recent trauma. Laboratory examinations revealed a white blood cell count of 11,070/mmc with 10,000/mmc neutrophils (90%) and 500/mmc Lymphocytes (4,5%), haemoglobin level of 10.3 g/dL, and platelet count of 215,000/mmc. The C-reactive protein (CRP) was 42 mg/dL (reference range 0,0–0,15). The patient was transferred to our hospital for evaluation of possible appendicitis. On admission, she appeared ill with low-grade fever (anti-inflammatory drug recently taken), a pulse rate of 120 beats/min, and a respiratory rate of 18–20 breaths/min. Physical examination revealed a strained abdomen with pain on palpation of the right iliac fossa. Musculoskeletal examination revealed restricted movement of the right hip with pain radiating to the lumbosacral region, but no warmth or erythema. The results of the remaining examinations were unremarkable. Laboratory examination results confirmed neutrophilic (9030/mmc) leukocytosis (10,770/mmc) with considerable elevation of inflammatory indices such as CRP 46.17 mg/dl (range 0,0–0,15 mg/dl), Procalcitonin 25.8 ng/ml (range 0,0–0,50 ng/ml); d-dimer, 6199 ng/ml (range, < 500 ng/ml) and fibrinogen level, 874 mg/dl (rang 150–400 mg/dl). The examinations showed renal insufficiency (creatinine 1,24 mg/dl, range 0,35 − 0,86 mg/dl); therefore, intravenous hydration was immediately initiated. Serum electrolyte, amylase, lipase, liver function tests, and urinalysis results were normal. Chest-X-ray did not reveal any pulmonary foci of infection and abdomen ultrasonography was unremarkable. So, we performed a Contrast-enhanced computed Tomography (CT) and a magnetic resonance imaging (MRI) of the right hip that revealed pyomyositis of the iliopsoas, piriformis, and internal shutter associated with collection of pus (abscess) between the muscular planes (Fig. 1).

We started intravenous broad-spectrum antibiotic therapy with ceftriaxone (100 mg/kg/day) and vancomycin (60 mg/kg/day). After 48 h, the blood culture was positive for Streptococcus pneumoniae, sensitive to penicillin, ceftriaxone, and clindamycin; therefore, the antibiotic therapy was switched to intravenous ceftriaxone only.

**Fig. 2:**
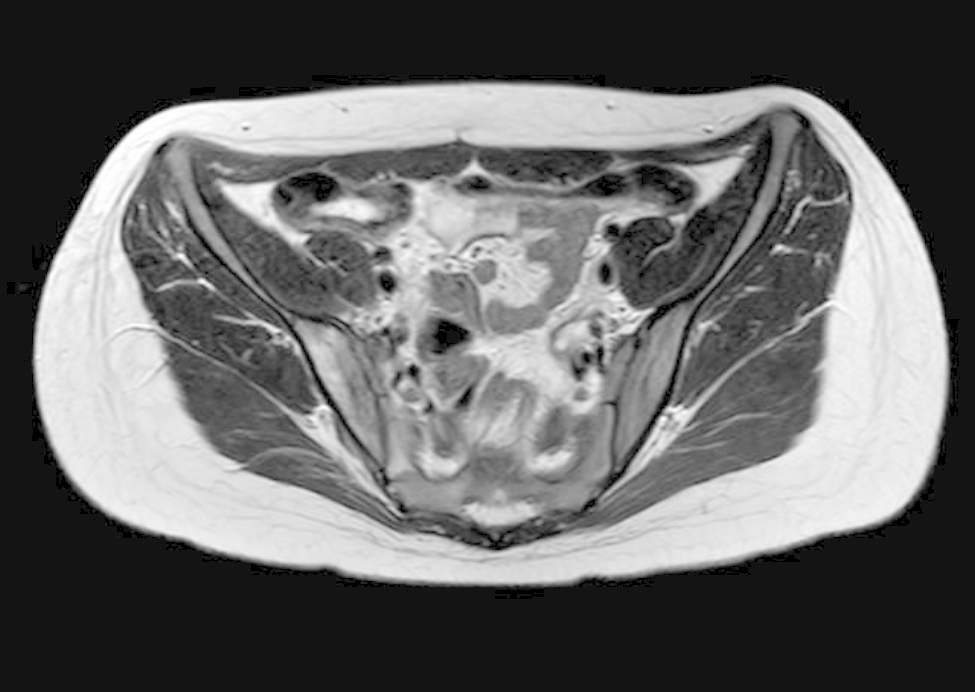
Magnetic Resonance Imaging after two months. we observed a completely resolution of muscles edema and abscess formation

After four days of hospitalization, the fever declined, and leg pain resolved after 14 days of recovery, without adverse events. Repeated skeletal muscular ultrasound on third, the seventh, the thirteenth and the twentieth hospital days showed progressive reduction of the abscess. During the recovery the pneumococcal urine antigen test resulted negative. Moreover, multiplex reverse-transcriptase polymerase chain reaction (RT-PCR) on nasopharyngeal swab for respiratory viruses and bacteria (including Mycoplasma pneumoniae, Chlamydophila pneumoniae, Legionella pneumoniae and Bordetella pertussis) was performed, with negative results. The child was discharged after 21 days of treatment with intravenous ceftriaxone and continued oral antibiotic therapy at home with oral amoxicillin to complete a total of 6 weeks of antibiotic therapy. Immunological studies revealed normal IgG, IgA, IgM, and IgG subclass values and normal classical and alternative pathway total complement function. MRI performed 2 months later showed complete resolution of the pyomyositis and abscess (Fig. 2).

## Methods

The studies included in this review were found after searching the PubMed database using the following search strings: “Myositis or Pyomyositis”, “Streptococcus Pneumoniae” and “Psoas Abscess” filtered by paediatric range. In total, 147 articles were identified. Of these, we excluded those unrelated to the purpose of our study and those with pyomyositis caused by pathogens other than Streptococcus Pneumoniae. The aim of this study is to analyse risk factors, clinical presentation, correct antibiotic therapy, and outcomes of pyomyositis caused by Streptococcus pneumoniae. To obtain a complete overview of the topic, we also examined the most recently available guidelines on antibiotic therapy and their bibliographic entries.

## Discussion and conclusions

Pyomyositis caused by Streptococcus pneumoniae is rare in pediatric patients [5]. Our literature review revealed nine cases of pneumococcal pyomyositis in children (< 15 years old), including our case report (Table 1). The youngest patient was 22 days old. In two cases, preceding trauma was documented (5–6). In three children, pyomyositis was associated with otitis media, pneumonia, and meningitis [7–9]. In one case, the patient had an immune defect (C3 complement deficiency) [9]. In five cases, Streptococcus pneumoniae was isolated from the abscess culture [6, 8–10] and in four cases from blood culture [5, 7, 11], including the present case report. The average white blood cell count was 25,72 × 109/L with a prevalence of neutrophils. The mean CRP was 24,72 mg/dl. More than one muscle was implicated in 33% of cases. The main diagnostic method used was MRI in six cases [5, 7–9, 11], following by ultrasound (4 cases, all associated with MRI)[7–9] and CT (3 cases)[6, 10, 12]. All patients were treated with antibiotics; in four cases, first- and third generation cephalosporins were used, followed by clindamycin (3 cases) and penicillin (3 cases). The mean duration of intravenous antibiotic therapy was 21 days. In 5 cases, antibiotic therapy was associated with drainage of abscess formation. None of the patients experienced any long-term complications with complete resolution. All data are summarized in Table 1.

**Table 1:** Cases of pyomyositis by Streptococcus Pneumonia in children in literature

Age (y)	22 days	8 Mo	2	3	4	12	13	13	12 (present report)
Sex	M	M	M	M	M	M	F	F	F
Preceding or association illness	None	Meningitis	Otitis Media	None	Pneumonia	None	Trauma	Trauma	None
Underling condition	None	None	None	None	C3 complement deficency	None	Unknown	None	None
Positive abscess colture	Yes	No	Yes	Yes	Yes	No	Yes	No	No
Positive blood colture	No	Yes	No	No	No	Yes	No	Yes	Yes
Muscles involved	Ileopsoas	Quadricipes femoris	Biceps Brachialis	Subscapularis, Gluteus minimus	Ileopsoas	Iliacus, Piriformis, Gluteus Maximum, gluteus medium	Ileopsoas	Ileopsoas	iliacus, piriformis, internal shutter
White blood cell (x10^9/L)	36,7	18,3	28		28	64		19,7	11,07
Neutrophilis (%)	Unknown	58	64		75	78			90
CRP (mg/dl)	26,4	35,8	4,43		22,9	16,83			42
Erythrocyte sedimentation rate (mm/h)						58		126	
Diagnostic Imaging	CT	Ultrasound, MRI	Ultrasound, MRI	CT	Ultrasound, MRI	MRI	CT	MRI	Ultrasound, MRI
Antibiotics treatment	Yes	Yes	Yes	Yes	Yes	Yes	Yes	Yes	Yes
Type of antibiotics	Ampicillin	Penicillin + Clindamycin	Cefuroxime + clindamycin	Cefazolin	Ampicillin	Clindamycin	Unknown	Cefazolin	Ceftriaxone
Duration of IV tratment (days)	28	10	30		21	21		21	21
Recovery	Yes	Yes	Yes		Unknown	Yes		Yes	Yes
Abscess drainage	Yes	No	Yes	Yes	Yes	No	Yes	No	No
Complication	None	None	None	None	None	None	None	None	None

The youngest patient was 22 days old. In two cases, preceding trauma was documented. In three children, pyomyositis was associated with otitis media, pneumonia, and meningitis. In one case, the patient had an immune defect (C3 complement deficiency. In five cases, Streptococcus pneumoniae was isolated from the abscess culture and in four cases from blood culture, including the present case report. The average white blood cell count was 25,72 × 109/L with a prevalence of neutrophils. The mean C-Reactive Protein was 24,72 mg/dl. More than one muscle was implicated in 33% of cases. The main diagnostic method used was Magnetic Resonance Imaging (MRI) in six cases, following by ultrasound (4 cases, all associated with MRI) and Computer Tomography (CT) (3 cases). All patients were treated with antibiotics; in four cases, first- and third generation cephalosporins were used, followed by clindamycin (3 cases) and penicillin (3 cases). The mean duration of intravenous antibiotic therapy was 21 days. In 5 cases, antibiotic therapy was associated with drainage of abscess formation. None of the patients experienced any long-term complications with complete resolution.

MRI (magnetic resonance imaging), CT (computed tomography), IV (intravenous), RCP (C-reactive-protein). Cases of pyomyositis by Streptococcus Pneumonia in children in literature

The present report emphasizes the challenges in the clinical diagnosis and management of pneumococcal pyomyositis in pediatric patients. The clinical presentation is similar to that of non-pneumococcal pyomyositis characterized by fever, swelling, and pain [1–4]; although some patients have symptoms correlated to other conditions (pneumonia, otitis media, meningitis) that could be the source of muscular infection [8]. These symptoms could mimic septic arthritis, osteomyelitis, deep vein thrombosis, and appendicitis, making it difficult to diagnose. Multifocal infections affecting more than one muscle can be present in 10–20% of cases [3], as in our clinical case.

Routing laboratory tests are not always specific and are rarely helpful in the diagnosis of pyomyositis [14]. Usually, neutrophilic leukocytosis associated with elevation of inflammatory markers such as CRP or Erythrocyte sedimentation rate is present during this disease [14]. It is important to obtain cultures (blood and, if possible, the involved site) every time pyomyositis is suspected to identify the infective microorganism and its susceptibility pattern. During recovery, it is important to evaluate the immune system of patients to determine the presence of predisposing conditions (not present in our case).

Once infection is suspected, various non-invasive diagnostic methods can be used to evaluate the diagnosis, such as ultrasound, computed tomography (CT), and MRI. Recent literature recommends the use of MRI imaging modality for establishing the diagnosis of pyomyositis as the first choice. Other techniques could also be useful for describing lesions [13]. Ultrasonography was not relevant in our clinical case. CT showed muscle inflammation and asymmetry associated with a hypodense collection. On the other hand, MRI revealed the extent of involvement and site of fluid collection. MRI is the gold standard because it can study adjacent structures, such as joints, bone, and soft tissue, and is therefore helpful in differentiating other pathological processes from pyomyositis, such as osteomyelitis, hematoma, or soft tissue tumor [14, 15]. Both CT and ultrasonography are less sensitive than MRI for detecting pyomyositis but may be helpful for percutaneous abscess drainage [15]. Ultrasonography can be useful for monitoring muscle inflammation during therapy (14–15).

The choice of treatment was correlated with the stage of the infection. Antibiotic therapy is the mainstay of Pneumococcal pyomyositis and most of patients could be successfully treated with intravenous administration of a single antibiotic [8]. Streptococcus Pneumoniae has gradually become more resistant to penicillin in European countries [8]. In our clinical case, we started empirical antibiotic therapy with double antibiotics (cephalosporin and glycopeptide) for the first 48 h, and then shifted to a single antibiotic (third-generation cephalosporin) to complete three weeks of intravenous antibiotic therapy. Therefore, based on antimicrobial susceptibility tests, we can consider other antibiotics, such as carbapenems, vancomycin, and clindamycin. The duration of therapy has not been established and can range from two to six weeks [15], depending on the patient’s response and clinical severity. Surgical drainage does not always seem necessary for cure. In many cases, despite abscess formation, neither surgical nor percutaneous drainage is performed. However, Streptococcus pneumoniae pyomyositis may be associated with muscle necrosis; therefore, if there is no improvement in clinical, laboratory, and imaging conditions, it is essential to drain the formation [13–15]. In our case, considering the prompt response to antibiotic therapy, drainage of the abscess muscles was not required.

In conclusion, pyomyositis caused by Streptococcus pneumoniae is a rare and potentially dangerous disease in children. In our clinical case, the early diagnosis was difficult. It is important, in case of suspicion, to perform laboratory examinations, including culture, to identify microorganisms and their susceptibility patterns. MRI remains the gold standard for identifying pyomyositis, and ultrasound could be useful for evaluating the response to antibiotic therapy. Our case confirmed that pyomyositis is not only an exclusive pathogen in tropical countries or immunosuppressed patients and may be caused by organisms other than Staphylococcus aureus.

## Electronic supplementary material

Below is the link to the electronic supplementary material.

Supplementary Material 1

## Data Availability

the datasets during and/or analysed during the current study available from the corresponding author on reasonable request.

## List of abbreviation

CRP: C-reactive protein
CT: Computed tomography
MRI: Magnetic resonance imaging
IV: Intravenous
RT-PCR: Reverse-transcriptase polymerase chain reaction
