# Supplementary material for: Pyomyositis associated with abscess formation caused by streptococcus pneumoniae in children: a case report and review of literature
Source: Ital J Pediatr. 2023 Jun 14;49:73. doi: 10.1186/s13052-023-01472-1 (PMC10268487; doi:10.1186/s13052-023-01472-1)
Supplement: Supplementary file 1 — Supplementary Material 1 [file 13052_2023_1472_MOESM1_ESM.pdf]

CARE case report guidelines

CARE Checklist of information to include when writing a case report

(cc) BY-NC-ND

Topic	Item	Checklist item description	Reported on Line
Title	1	The diagnosis or intervention of primary focus followed by the words "case report"	Lines 1-2
Key Words	2	2 to 5 key words that identify diagnoses or interventions in this case report, including "case report"	Line 11
Abstract (no references)	3a	Introduction: What is unique about this case and what does it add to the scientific literature?	Lines 18-21
	3b	Main symptoms and/or important clinical findings	Lines 22-25
	3c	The main diagnoses, therapeutic interventions, and outcomes	Lines 25-32
	3d	Conclusion—What is the main "take-away" lesson(s) from this case?	Lines 34-38
Introduction	4	One or two paragraphs summarizing why this case is unique (**may include references**)	Lines 42-51
Patient Information	5a	De-identified patient specific information.	Line 55
	5b	Primary concerns and symptoms of the patient.	Lines 55-57
	5c	Medical, family, and psycho-social history including relevant genetic information	Lines 55-60
	5d	Relevant past interventions with outcomes	Lines 56-60
Clinical Findings	6	Describe significant physical examination (PE) and important clinical findings.	Lines 60-64
Timeline	7	Historical and current information from this episode of care organized as a timeline	Lines 55-64
Diagnostic Assessment	8a	Diagnostic testing (such as PE, laboratory testing, imaging, surveys).	Lines 65-70
	8b	Diagnostic challenges (such as access to testing, financial, or cultural)	Lines 70-73
	8c	Diagnosis (including other diagnoses considered)	Lines 72-73
	8d	Prognosis (such as staging in oncology) where applicable	
Therapeutic Intervention	9a	Types of therapeutic intervention (such as pharmacologic, surgical, preventive, self-care)	Lines 77-88
	9b	Administration of therapeutic intervention (such as dosage, strength, duration)	Lines 77-88
	9c	Changes in therapeutic intervention (with rationale)	Lines 85-88
Follow-up and Outcomes	10a	Clinician and patient-assessed outcomes (if available)	
	10b	Important follow-up diagnostic and other test results	Lines 97-98
	10c	Intervention adherence and tolerability (How was this assessed?)	Lines 90-98
	10d	Adverse and unanticipated events	Line 94
Discussion	11a	A scientific discussion of the strengths AND limitations associated with this case report	Lines 111-125
	11b	Discussion of the relevant medical literature **with references**.	Lines 128-151
	11c	The scientific rationale for any conclusions (including assessment of possible causes)	Lines 15-166
	11d	The primary "take-away" lessons of this case report (without references) in a one paragraph conclusion	lines 168-174
Patient Perspective	12	The patient should share their perspective in one to two paragraphs on the treatment(s) they received	Lines 90-98
Informed Consent	13	Did the patient give informed consent? Please provide if requested	Yes ☑ No ☐
